# Plasmonic Sensor Based on Interaction between Silver Nanoparticles and Ni^2+^ or Co^2+^ in Water

**DOI:** 10.3390/nano8070488

**Published:** 2018-07-02

**Authors:** Federico Mochi, Luca Burratti, Ilaria Fratoddi, Iole Venditti, Chiara Battocchio, Laura Carlini, Giovanna Iucci, Mauro Casalboni, Fabio De Matteis, Stefano Casciardi, Silvia Nappini, Igor Pis, Paolo Prosposito

**Affiliations:** 1 Department of Industrial Engineering and INSTM, University of Rome, Tor Vergata, via del Politecnico 1, 00133 Rome, Italy; federico.mochi@uniroma2.it (F.M.); luca.burratti@uniroma2.it (L.B.); casalboni@uniroma2.it (M.C.); fabio.dematteis@uniroma2.it (F.D.M.); 2 Center for Regenerative Medicine, University of Rome Tor Vergata, Via Montpellier 1, 00133 Rome, Italy; 3 Department of Chemistry, University of Rome Sapienza, Rome, P.le A. Moro 5, 00187 Rome, Italy; ilaria.fratoddi@uniroma1.it; 4 Department of Sciences, Roma Tre University of Rome Via della Vasca Navale 79, 00146 Rome, Italy; chiara.battocchio@uniroma3.it (C.B.); laura.carlini@uniroma3.it (L.C.); giovanna.iucci@uniroma3.it (G.I.); 5 National Institute for Insurance against Accidents at Work (INAIL), Department of Occupational and Environmental Medicine, Epidemiology and Hygiene, 00078 Monte Porzio Catone, Italy; s.casciardi@inail.it; 6 IOM-CNR Laboratorio TASC, SS 14, km 163,5 Basovizza, I-34149 Trieste, Italy; nappini@iom.cnr.it; 7 Elettra-Sincrotrone Trieste S.C.p.A., SS 14, km 163.5 Basovizza, I-34149 Trieste, Italy; igor.pis@elettra.eu

**Keywords:** silver nanoparticles, surface plasmon resonance, heavy metal ions sensing, Ni^2+^ sensing, Co^2+^ sensing, water pollution, optical sensors

## Abstract

Silver nanoparticles capped with 3-mercapto-1propanesulfonic acid sodium salt (AgNPs-3MPS), able to interact with Ni^2+^ or Co^2+^, have been prepared to detect these heavy metal ions in water. This system works as an optical sensor and it is based on the change of the intensity and shape of optical absorption peak due to the surface plasmon resonance (SPR) when the AgNPs-3MPS are in presence of metals ions in a water solution. We obtain a specific sensitivity to Ni^2+^ and Co^2+^ up to 500 ppb (part per billion). For a concentration of 1 ppm (part per million), the change in the optical absorption is strong enough to produce a colorimetric effect on the solution, easily visible with the naked eye. In addition to the UV-VIS characterizations, morphological and dimensional studies were carried out by transmission electron microscopy (TEM). Moreover, the systems were investigated by means of dynamic light scattering (DLS), Fourier-transform infrared spectroscopy (FTIR), X-ray photoelectron spectroscopy (XPS), and high-resolution X-ray photoelectron spectroscopy (HR-XPS). On the basis of the results, the mechanism responsible for the AgNPs-3MPS interaction with Ni^2+^ and Co^2+^ (in the range of 0.5–2.0 ppm) looks like based on the coordination compounds formation.

## 1. Introduction

The huge development of materials science, nanoscience, and nanomaterials technology has led to the synthesis and engineerization of several nanostructures (metallic and non) used in different fields such as biomedicine [1,2], biotechnology [3,4], energy [5,6,7,8], optics, and optoelectronics [9,10,11,12,13,14,15,16]. Recently, sensors based on different nanosized materials have been developed, achieving a high sensitivity and selectivity [17,18,19,20,21,22,23,24,25,26].

In this framework, metallic nanoparticles cover an important role for the easy synthesis, the low costs, and for the possibility to accomplish specific external functionalization to respond selectively to specific analytes. The reduced dimensions of the metallic structures confer them unique physical, chemical, mechanical, optical, magnetic, and catalytic properties [27,28,29,30,31,32]. In particular, the small dimensions allow optical properties, which make metallic nanoparticles interesting for optical spectroscopy applications such as surface enhanced Raman spectroscopy (SERS) and surface plasmon resonance (SPR). The latter phenomenon occurs when an electromagnetic radiation of a certain wavelength, exciting the nanoparticle, causes the oscillation of free electrons in the conductive band. As a result, the optical absorption presents an intense and well-shaped SPR, usually in the visible region. The wavelength of the SPR peak strongly depends on the type of metal, particle dimension, shape, and chemical environment. Another important aspect of these systems is their high surface to volume ratio, which confers them a very high reactivity with the surroundings as well as the possibility to modify their external surfaces with an appropriate surface chemistry. On this basis, many metal nanoparticles-based sensors have been studied and developed [33,34,35,36,37]. The main application of such optical sensors is the detection of heavy-metals, which have long been known to be harmful for the environment and toxic for human health, above very small concentrations, a few ppm or lower, as reported in the literature [38,39,40] and by the Guidelines for Drinking-Water Quality by the World Health Organization (WHO) [41].

The current state of the art for the detection of heavy metal ions is based on complex and time consuming techniques, such as high performance liquid chromatography (HPLC), atomic fluorescence spectroscopy (AFS), flame atomic absorption spectroscopy (FAAS), and graphite furnace atomic absorption spectroscopy (GFAAS) [42,43]. All of these methods are very sensitive and reliable, but they have also some disadvantages, as the complexity and the high instrumentation costs, together with the requirement of highly skilled operators. For all these reasons, the scientific community is currently working on innovative, simple, and low cost heavy metal ions sensors. In particular, the ones based on optical and colorimetric techniques have received great attention, as they can offer high selectivity, stability, intrinsic operational simplicity, and immunity against electrical disturbance. In addition, they are extremely attractive as they are based on simple and low cost materials, are very easy to use, are portable, and need simple and cheap set ups, offering, at the same time, high sensitivity and selectivity.

In the present work, AgNPs-3MPS were synthetized and their interaction with heavy metal ions was studied using different techniques. The system presents a strong sensitivity to Ni^2+^ and Co^2+^ ions, showing a consistent change in the SPR as a function of the ion concentration, resulting in a colorimetric change of the solution. The morphological and dimensional characterizations of the AgNPs-3MPS (average size and shape) before and after the interaction with Ni^2+^ and Co^2+^ were obtained by TEM studies. Moreover, the system was studied by means of different techniques, such as dynamic light scattering (DLS), UV-VIS, FTIR, and high-resolution X-ray photoelectron spectroscopy (HR-XPS), in order to understand the mechanism of SPR sensing.

## 2. Materials and Methods

### 2.1. Materials

Silver nitrate (AgNO_3_, 99.5%, Sigma-Aldrich, St. Louis, MO, USA) and sodium borohydride (NaBH_4_, 98%, Sigma-Aldrich, St. Louis, MO, USA), were used for the synthesis of the nanoparticles. 3-mercapto-1propanesulfonic acid sodium salt (C_3_H_7_S_2_O_3_Na, 3MPS, Sigma Aldrich, 98%) was used as a capping agent. For the sensitivity measurement to the different ions, we used the following salts: Mg(ClO_4_)_2_, KClO_4_, NaClO_4_, Ca(ClO_4_)_2_, Pb(NO_3_)_2_, Cd(NO_3_)_2_, FeCl_3_ 6H_2_O, Cu(NO_3_)_2_, NiCl_2_ 6H_2_O, and CoCl_2_ 6H_2_O. For all of the solutions, we used deionized water (electrical conductivity less than 1 μΩ/cm at room temperature) obtained from a Millipore Milli-Q water purification system. All of the reagents were purchased from Sigma Aldrich and were used without further purification.

### 2.2. Synthesis and Characterization of AgNPs

The AgNPs were prepared by a wet reduction of silver nitrate in the presence of sodium borohydride. The thiol (3MPS) was subsequently added and it capped the silver nanoparticles. The details of this procedure were reported elsewhere [44]. The morphological characterization was accomplished with a TEM, FEI TECNAI 12 G2 (120 KeV) apparatus, equipped with an energy filter (GATAN GIF model) and a Peltier cooled SSC (slow scan charged coupled device) multiscan camera (794 IF model). A droplet of AgNPs water solution was placed on a copper TEM grid (mesh 400) coated with ultrathin carbon support. The UV-VIS spectra of water suspensions were collected using a Perkin-Elmer Lambda 19 spectrophotometer. The DLS measurements on the AgNPs colloidal suspensions (0.2 mg/mL) at T = 25.0 ± 0.2 °C were performed by the Malvern Zetasizer Nanoseries instrument (Malvern, UK), as reported in previous studies [45]. The ζ-potential was calculated from the measured electrophoretic mobility by means of the Smolukovsky equation [46]. Reflection absorption infrared spectroscopy (RAIRS) analysis was performed by means of a VECTOR 22 (Bruker, Billerica, MA, USA) FTIR interferometer equipped with a deuterated-triglycine sulfate detector (DTGS detector), and operating in the wavenumber range 400–4000 cm^−1^. The measurements were carried out by means of a Specac Monolayer/grazing angle accessory GS19650, operating at 70° incidence. The samples were prepared as thin films by solvent evaporation on Ti substrates from the mother solution; a clean Ti surface was used to record the background.

The HR-XPS experiments were carried out at the BACH (Beamline for Advanced DiCHroism) line at the ELETTRA synchrotron facility in Trieste (Italy) [47], to probe the nature of the interactions at the AgNPs/organic ligands interface and the formation of Ni^2+^ or Co^2+^ coordination compounds. All of the samples were deposited by means of a drop casting procedure on a silicon wafer substrate (TiO_2_/Si (111 plane)). The XPS data were collected in a fixed analyzer transmission mode (pass energy = 30 eV). Photon energies (PE) of 380 eV were used for C1s and S2p spectral regions, with an energy resolution ΔE = 0.2 eV, and a PE of 1050 eV was selected to acquire Ag3d, O1s, Ni2p, and Co2p core levels spectra, with an energy resolution ΔE = 0.3 eV. The aliphatic C1s signal and metallic Ag3d_5/2_ signals were used for the energy scale calibration. The XPS data analysis was performed via the curve-fitting of S2p, Ag3d, Ni2p, and Co2p experimental spectra, using a combination of Voigt shaped peaks, after the subtraction of a Shirley background. The S2p3/2-S2p1/2, Ag3d_5/2_-Ag3d_3/2_, Ni2p_3/2_-Ni2p_1/2_, and Co2p_3/2_-Co2p_1/2_ doublets were fitted using the same full width half maximum (FWHM) for the two spin-orbit components of the same signal, a spin-orbit splitting of 1.20 eV for S2p, 6.00 eV for Ag3d, 3.30 eV, 17.27 eV for Ni2p, and 14.97 eV for Co2p, and the branching ratios S2p_3/2_/S2p_1/2_ = 2, Ag3d_5/2_/Ag3d_3/2_ = 3/2, Ni2p_3/2_/Ni2p_1/2_ = 2, Co2p_3/2_/Co2p_1/2_ = 2 have been detected, respectively. For the S2p XPS spectra, many chemically different species of the same element were identified and the same FWHM value was used for all of the individual photoemission bands, in order to reduce the number of refinement parameters, and then improving the reliability of the results. In the Co2p and Ni2p spectral regions, shake-up satellites appear nearby the main photoelectron signals (at higher binding energy (BE)), as expected for the transition metals ions [48]; to fit satellite signals, variable FWHM and branching ratios were used, accordingly to the literature [49,50].

### 2.3. Sensing

The AgNPs-3MPS contained in a fixed volume of water (typically 0.014 mg in 1 mL) were added to a fixed volume of water solution containing the heavy metal ions at specific concentration (typically in 1 mL). After five minutes of interaction of the nanoparticles with the metal ions, the optical absorption spectra and the respective DLS measurements were collected. The response to several metal ions was tested by UV-VIS spectroscopy.

## 3. Results and Discussion

**Figure 1:**
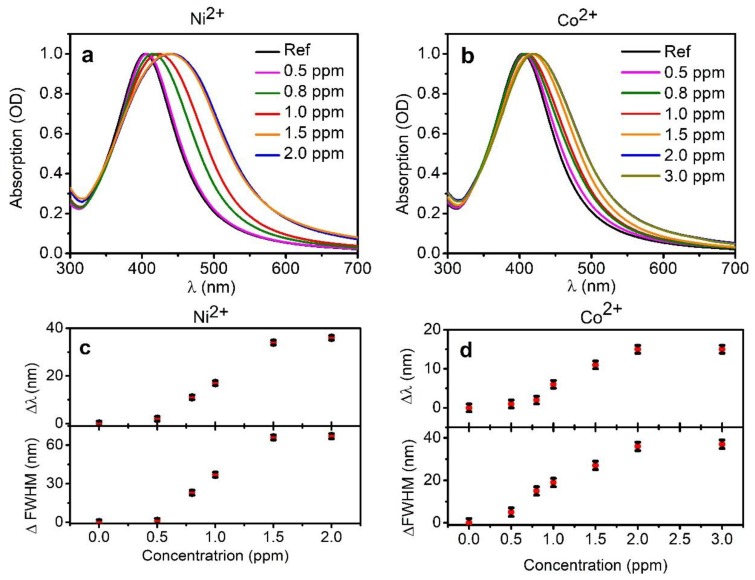
Optical absorption bands for Ni^2+^ (**a**) and Co^2+^ (**b**) as a function of the ion concentration (reported in the figure labels); variation of Δλ and variation of the full width at half maximum (ΔFWHM) of Ni^2+^ (**c**) and Co^2+^ (**d**), as a function of the ion concentration.

Figure 1 reports the SPR absorption band of the AgNPs-3MPS (reference solution) and the optical features of the same solution with a different concentration of nickel and cobalt ions, up to 2.0 ppm for nickel (a) and up to 3.0 ppm for cobalt (b), respectively. A red shift with the increasing concentration of ions is observed for both of the ions as well as a broadening of the SPR band. Figure 1c,d show the wavelength shifts (Δλ) and the variation of the full width at half maximum (ΔFWHM) of the Ag absorption band for increasing Ni^2+^ and Co^2+^ concentrations. We decided to report the FWHM of the absorption band, as it gives a qualitative idea of the broadening of the nanoparticles dimension distribution before and after the interaction with ions. Both of the figures show a saturation effect, represented by the plateau for high ion concentrations, 2 ppm for Ni^2+^ and 3ppm for Co^2+^. The saturation effect is reached at different ions concentration for nickel and cobalt.

The interaction of the Ni^2+^ ions with AgNP-3MPS causes a stronger modification of the shape and wavelength peak of the SPR with respect to presence of Co^2+^. In particular, Δλ is 36 nm for 2.0 ppm of Ni^2+^, while only 17 nm for 3.0 ppm of Co^2+^. A similar behavior was found for ΔFWHM, 67 nm for 2.0 ppm of nickel and 36 nm for 3.0 ppm of cobalt. Fitting those curves with a sigmoidal Richards function (y = a × (1 + (d − 1) × exp(−k × (x − xc)))^(1/(1−d))^), it is possible to obtain a correlation between Δλ and ΔFWHM with concentration. The fitting parameters of the function varies for each system (see supporting information Figure S1 for fitting graphics and table of parameters).

**Figure 2:**
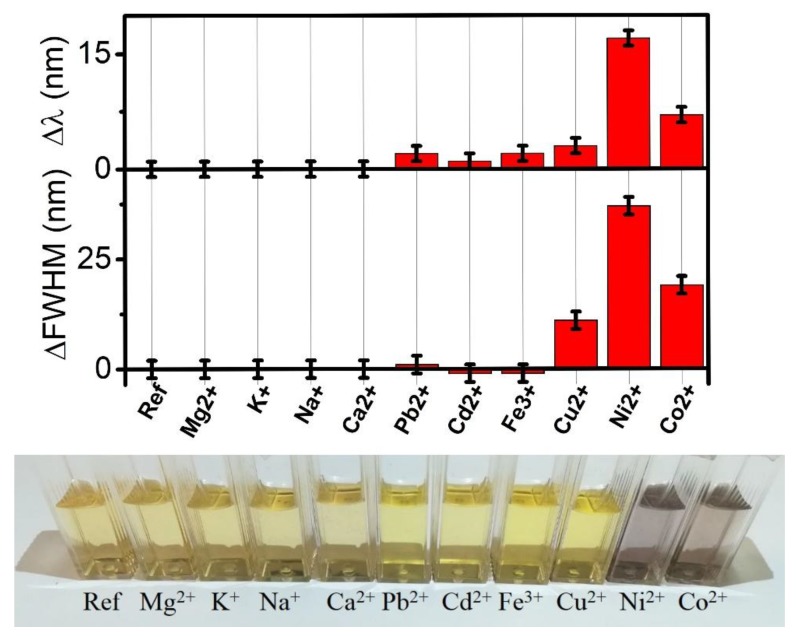
Δλ and ΔFWHM of the AgNPs-3MPS solution with 1 ppm of different metal ions. Image of the colorimetric aspect of 1.0 ppm solution of different ions.

We checked the selectivity of the colloidal system to other ions. Figure 2 (upper part) shows the variation of the optical plasmonic characteristics (peak wavelength and shape) of the AgNP-3MPS solution with 1.0 ppm of the specific ions listed in the figure. The response to the non-toxic ions such as Mg^2+^, K^+^, Na^+^, and Ca^2+^, and to the toxic agents such as, Pb^2+^, Cd^2+^, and Fe^3+^, is clearly not significant. Cu^2+^ shows a small modification of the shape (ΔFWHM) of the SPR and a negligible change of the maximum peak wavelength. The most relevant differences were measured for Ni^2+^ and Co^2+^. Figure 2 (lower part) reports a picture of the AgNPs-3MPS solution treated with a fixed amount (1 ppm) of different ions; the colorimetric changes can be easily appreciated also by naked eye. The different optical response to nickel and cobalt ions can be exploited for a selective detection of these specific species.

The DLS measurements were carried out, showing an increase of the average hydrodynamic diameter (<2R_H_>) of the AgNPs-3MPS, after interaction with the Co^2+^ or Ni^2+^ ions (see supporting information Figures S2–S4). Table 1 shows the dimension of NPs as a function of the increasing concentration of Ni^2+^ ions, from (8 ± 3) nm for reference, that is, AgNPs-3MPS alone, to (10 ± 1) nm for 0.5 ppm, to (43 ± 4) nm for 1.0 ppm, to (1110 ± 100) nm for 2.0 ppm of Ni^2+^ concentration. Moreover, the ζ potential changes in presence of Ni^2+^ as follows: AgNPs-3MPS showed ζ potential of −44 mV, while in presence of 1.0 ppm of Ni^2+^, the ζ potential becomes −27 mV, indicating an effective interaction of the positive ions with NPs. In Table 1, similar results are also reported for increasing the concentration of Co^2+^ ions from 0.5 to 2.0 ppm. In this case, the particles’ dimensions have a fairly similar trend of aggregation, up to become microsized, polydispersed, and instable in presence of 2 ppm of metal ion. In fact, the ζ potential evidences the interaction and becomes less negative or completely instable as in the case of 2 ppm of Co^2+^, reported in Table 1.

Therefore, we can observe that the stable water suspension of AgNPs readily aggregate in the presence of Ni^2+^ or Co^2+^, owing to cooperative effect of electrostatic and coordination interactions. The interaction between the AgNPs-3MPS and Co^2+^ or Ni^2+^ ions does not produce significant modification in the FTIR spectra (see supporting information Figure S5); the shape and position of the characteristic peaks related to AgNPs-3MPS undergo only a slight change.

**Figure 3:**
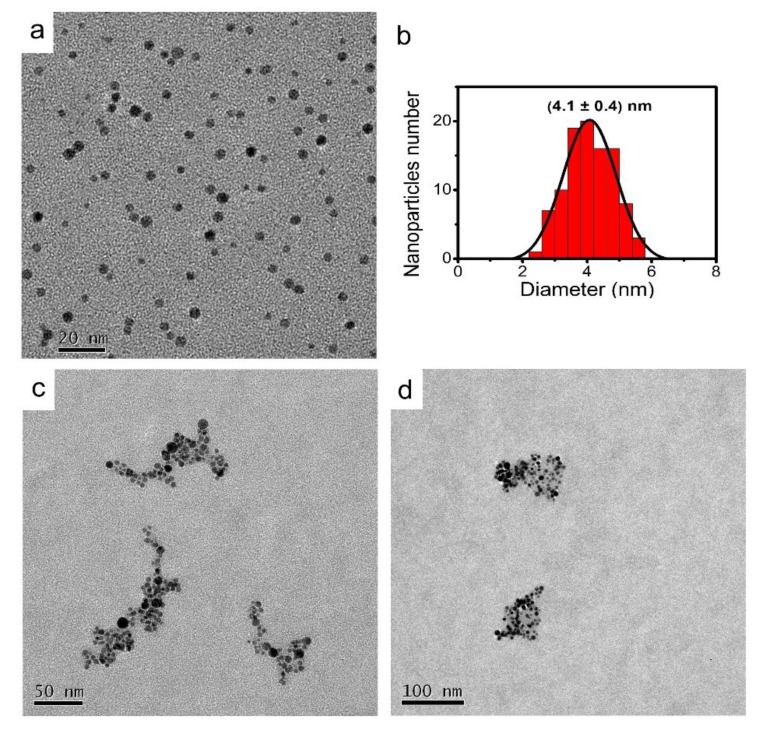
TEM image of AgNPs-3MPS (**a**); histogram of the average dimensions of the nanoparticles obtained by the analysis of 100 particles (**b**); scale bars are reported in the lower part of the pictures. TEM image of AgNPs-3MPS with 1.0 ppm of Ni^2+^ (**c**); and TEM image of AgNPs-3MPS with 1.0 ppm of Co^2+^ (**d**).

The Figure 3a reports a TEM image of the AgNPs-3MPS showing the shape and dimension of the nanoparticles. A spherical shape of the NPs can be appreciated together with an almost homogeneous distribution of the dimensions (see also supporting information Figure S6). The analysis of TEM images, based on a statistic on one hundred nanoparticles, reveals an average dimension of (4.1 ± 0.4) nm. This value is in agreement with the average diameter measured by DLS of (8 ± 3) nm, in fact, this last technique measures the hydrodynamic diameter of the nanoparticle and not the bare particle dimension, resulting in a greater diameter. Figure 3b shows the histogram of the average dimensions of the particles obtained analyzing 100 particles. Figure 3c,d show the typical shape of the AgNPs-3MPS aggregates for 1 ppm of Ni^2+^ and Co^2+^, respectively.

The dimensions and shapes are quite different. For cobalt ions, the aggregates are smaller and more regular in shape, with respect to the nickel ones. This indicates the interaction occurring and the possible formation of coordination compounds of nickel and cobalt ions with AgNPs-3MPS in analogy to literature data [51]. This behavior is also strongly supported by the HR-XPS characterization (vide infra). Moreover, considering the electrochemical analogies between Ni^2+^ and Co^2+^ ions, with standard reduction potentials quite close (−0.23 V and −0.28 V for Ni^2+^/Ni° and Co^2+^/Co°, respectively), it is possible to explain the similar behavior of the two ions in solution.

Figure S7 in supporting information reports a high resolution TEM image of a single nanoparticle presenting a greater radius with respect to the average value, but showing the good crystallinity of the material. The measured lattice parameter is 0.24 nm (111 plane), as reported in the figure, which is in close agreement with the lattice parameter of the silver nanoparticles reported in the literature [52,53].

To assess the silver nanoparticles molecular stability and to gain insights in the AgNPs-3MPS/ions interaction, HR-XPS measurements were carried out on thiol-functionalized silver NPs with Ni or Co ions-water solution, at concentrations 0.5, 1.0, 1.5, and 2.0 ppm (concentrations of 1.0 ppm for both ions will be here reported as examples), collecting the data at the C1s, O1s, S2p, Ag3d, Ni2p, or Co2p core levels. The AgNPs-3MPS were also measured as a reference for the data analysis discussion.

First of all, the Ag3d spectra were collected and analysed with the aim of probing the metal nanoparticles stability and to gain information about their dimensions. The XPS data and best fits for the Ag3d core levels are shown in supporting information Figure S8. The main quantitative results are summarized in Table 2.

**Figure 4:**
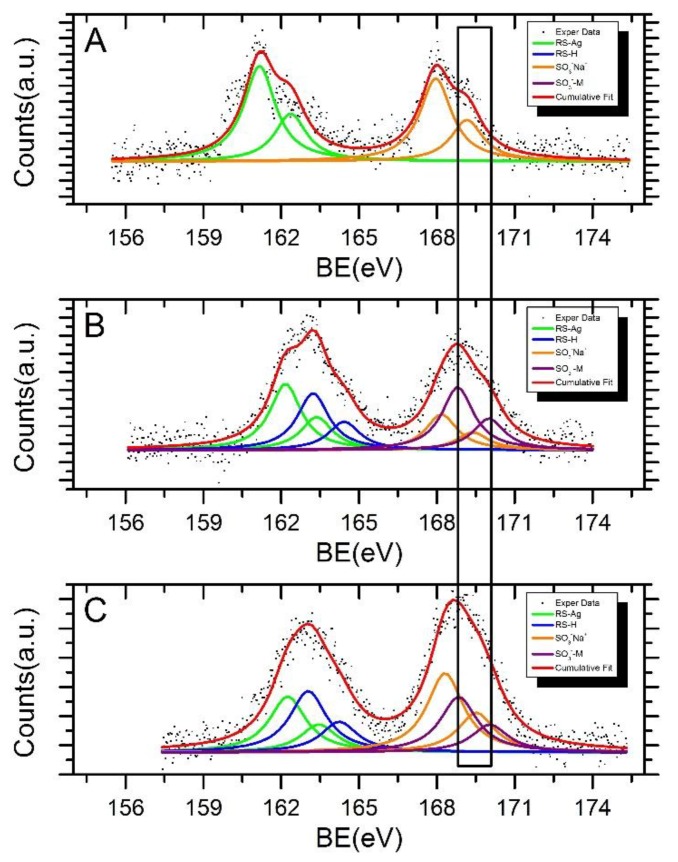
S2p X-ray photoelectron spectroscopy (XPS) spectra of samples AgNPs-3MPS (panel **A**); AgNPs-3MPS + Ni^2+^ 1 ppm (panel **B**); and AgNPs-3MPS + Co^2+^ 1 ppm (panel **C**).

Following a curve fitting procedure, two spin-orbit pairs were individuated in the Ag3d XPS spectra (see supporting information Figure S8). The main Ag3d_5/2_ components are centered around 368.1–368.2 eV binding energy (BE) value, and correspond to metallic silver atoms in the nanoparticles core, as expected and extensively reported in the literature for functionalized Ag nanoparticles [54]. The second spin-orbit pairs of small intensity at higher BE values (Ag3d_5/2_ BE of about 369.1–369.3 eV) are attributed to the more oxidized surface Ag bonded with an organic structure, as suggested in the literature for analogous systems [55]. The difference in the BE between the first Ag3d_5/2_ and the second Ag3d_5/2_ component is of about 1 eV for all of the investigated samples. The atomic percents detected for the metal associated to the covalent bond with sulphur (see Table 2) are consistent with the nanoparticles dimension indicated by TEM. Indeed, the percent of metal atoms located on the nanoparticle surface suggests a high surface to volume ratio, as expected for particles below 5 nm diameter [56]. To investigate the chemical environment at the interface between the AgNPs and 3MPS thiol, and to probe the hypothesized formation of the Ni and Co coordination compounds, the HR-XPS S2p core levels signals were collected and analysed. The spectra and complete BE, FWHM, atomic ratio, and assignments for the functionalized nanoparticles are reported in Table 3 and Figure 4, respectively.

**Figure 5:**
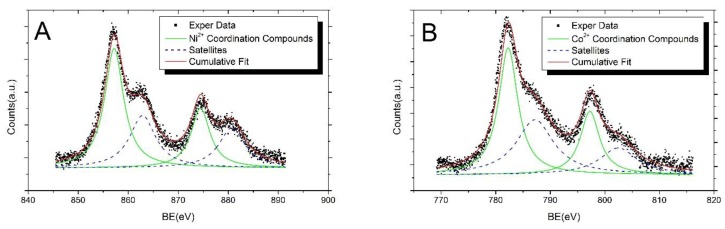
Ni2p and Co2p core levels spectra of thiol-functionalized AgNPs with 1.0 ppm of nickel (panel **A**) and 1.0 ppm of cobalt (panel **B**).

The S2p spectra analysis carried out on functionalized AgNPs in the presence of Ni and Co ions points out four distinct chemical states for S, the spin-orbit doublets at lower BE values (from literature BE S2p_3/2_ components around 161–162 eV) can be associated to RS-Ag bonded thiol [57,58]; more in detail, S2p_3/2_ components at about 161 eV and 162 eV are attributed to sulphur atoms covalently bonded to metals in a monolayer or sub-monolayer regime, with the S atom in two different hybridizations, sp (around 161 eV) and sp3 [59]. The S2p_3/2_ signal occurring at nearly 163.0–163.2 eV BE is due to sulphur atoms of the thiol moiety of physisorbed 3MPS molecules; conversely, the spin-orbit pair at higher BE values (S2p_3/2_ components BE close to 168.0–168.3 eV) is related to S in sulfonates functional groups (–SO_3_^–^), and finally, the S2p_3/2_ signal at BE = 168.8 eV can be attributed to sulfonate groups coordinating nickel or cobalt ions. It is noteworthy the absence of the spin-orbit pair at higher BE values for the pristine AgNPs-3MPS (see Table 3 and Figure 4), supporting the hypothesis that the S2p component around 168.8 is actually indicative for the formation of Ni^2+^ and Co^2+^ coordination compounds. The FWHM for XPS S2p signals was always found to be between 1.5 and 1.7 eV, consistent with literature reports on analogous systems [56,60]. The fraction of S atoms in the sulfonate groups (both free and coordinated to metal ions), with respect to the chemisorbed and physisorbed thiol moieties, is 0.8:1 for AgNPs-3MPS + Ni^2+^ and 1.1:1 for AgNPs-3MPS + Co^2+^, and the atomic ratio between the sulfonates and thiols signal is 0.9:1 for the reference AgNPs-3MPS sample as well. Taking into account the statistic error in semi-quantitative XPS analysis, which is of about 5% of the estimated value [61], the observed experimental atomic ratios are in good agreement with the theoretical ones, based on the 3MPS chemical structure, confirming the molecule integrity for all of the samples. Moreover, the relative percent of S atoms involved in the coordination compounds formation, with respect to the sulfonate groups, can be qualitatively estimated by comparing the intensities of the metal-coordinated sulfur signals, with respect the –SO_3_^–^ signals. The fraction of atoms in the –SO_3_^–^ + M^2+^ (where M^2+^ is Ni^2+^ or Co^2+^, according to the sample) configuration, with respect the –SO_3_^–^, is 1.8:1 for the AgNPs-3MPS + Ni^2+^ sample and 0.7:1 for the AgNPs-3MPS + Co^2+^ sample, indicates that a higher number of Ni ions coordinates the NPs ligands, as already suggested by the larger aggregates of AgNPs-3MPS found in the TEM measurements. In Table 4 and Figure 5, the Ni2p and Co2p XPS data analysis results are reported. It is known that nickel and cobalt can form a variety of complexes in different stereogeometries containing the metal ions in different oxidation states. The variation of binding energies and shake-up satellites spectral behavior are a function of stereochemistry, magnetic properties, and ligand surroundings. The fitting procedure applied to analyse the Ni2p spectrum collected on the AgNPs-3MPS + Ni^2+^ sample (1.0 ppm) allowed identifying the presence of one spin-orbit pair and two shake-up satellites in both 2p levels (see Panel A in Figure 5). The Ni2p_3/2_ signal at BE = 856.2 eV can be associated with the Ni^2+^ oxidation state in the nickel coordination compounds [49]. Likewise, the Co2p data analysis carried out for the AgNPs-3MPS + Co^2+^ (1.0 ppm) sample led to individuate a single pair of spin-orbit components with the Co2p_3/2_ main component at about 782 eV in binding energy, as expected for the Co^2+^ oxidation state in cobalt coordination compounds [48,50].

**Figure 6:**
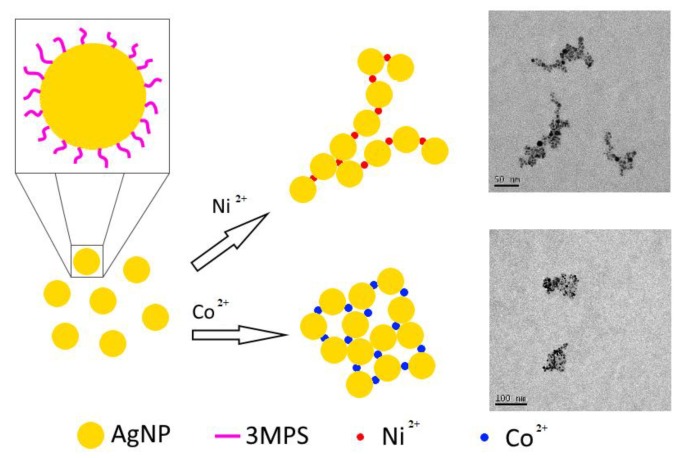
Scheme illustrating the different shape of the aggregates in presence of nickel and cobalt ions.

In conclusion, the HR-XPS analysis confirms the effective thiol-functionalization of the silver nanoparticles, without molecular degradation, and supports the hypothesis of the formation of the Ni^2+^ and Co^2+^ coordination compounds in the presence of nickel and cobalt ions in water. For the sake of clarity, we reported a very simple scheme illustrating the formation of the different aggregates in presence of the two ions, in Figure 6.

The XPS experimental results are also consistent with the NPs size, evidencing a high surface to volume atomic ratio, confirmed by the TEM measurements. The sensitivity limit measured in this work, of the order of 500 ppb for both Ni^2+^ and Co^2+^ ions, is coherent with recent works exploiting similar detection systems, reported in literature. For nickel sensing, Y. Shang at al. achieved a sensitivity of 14 ppb [62] and Li et al. of 4400 ppb [51]; while for cobalt, F. Zhang et al. reported values of 18 ppb [63] and V.N. Mehta et al. of 800 ppm [64]. The different sensibilities are clearly related to the external functionalization and to the complexity of the detection systems [65], however, they are in a range very similar to our results. In addition, it has to be underlined that our system is extremely easy to synthesize, even in large amounts. The obtained results are precious and helpful for a deep understanding of the detection mechanism, which is essential to develop simple and economic systems for a high precision colorimetric detection of heavy metal ions in water.

## 4. Conclusions

Optical sensor for Ni^2+^ and Co^2+^ ions in a water solution based on capped silver nanoparticles has been prepared and tested. The working principle of the sensor system is a change of the shape and the maximum wavelength of the surface plasmon resonance band. The AgNPs capped with 3MPS show selectivity for the two specific ions in water, Co^2+^ and Ni^2+^, and a good sensitivity up to 500 ppb. Careful analysis based on DLS, FTIR, and HR-XPS spectroscopies were performed together with TEM characterization, in order to understand the mechanism of SPR sensing. By the analysis, the mechanism responsible of the sensitivity of the AgNPs-3MPS seems based on the formation of the coordination compounds of Ni^2+^ and Co^2+^.

## Figures and Tables

**Table 1:** Dynamic light scattering (DLS) measurements of AgNPs-3MPS before and after interaction with the ions–water solution at specific concentrations. In the table, the average diameter and the ζ potential are reported. Errors quote the standard deviation of at least three independent measurements.

-	<2R_H_> (nm)	ζ Potential (mV)
AgNPs-3MPS alone	8 ± 3	−44 ± 5
AgNPs-3MPS + Ni^2+^ (0.5 ppm)	10 ± 1	−40 ± 6
AgNPs-3MPS + Ni^2+^ (1.0 ppm)	43 ± 4	−27 ± 10
AgNPs-3MPS + Ni^2+^ (2.0 ppm)	1110 ± 100	−13 ± 5
AgNPs-3MPS + Co^2+^ (0.5 ppm)	55 ± 5	−40 ± 14
AgNPs-3MPS + Co^2+^ (1.0 ppm)	76 ± 9	−22 ± 15
AgNPs-3MPS + Co^2+^ (2.0 ppm)	1436 ±108	-

**Table 2:** High-resolution X-ray photoelectron spectroscopy (HR-XPS) Ag3d_5/2_ metal core levels data collected on pristine AgNPs-3MPS and AgNPs-3MPS, in presence of 1 ppm of Ni and Co ions (binding energy (BE), full width half maximum (FWHM), atomic ratio, and assignments).

Sample	Signal	BE (eV)	FWHM (eV)	* I Ratio	Assignments
AgNPs-3MPS	Ag3d_5/2_	368.2	1.1	85%	Ag(0)
	Ag3d_5/2_	369.1	“	15%	Ag(δ^+^)
AgNPs-3MPS + Ni^2+^ (1.0 ppm)	Ag3d_5/2_	368.1	1.2	78%	Ag(0)
	Ag3d_5/2_	369.1	“	22%	Ag(δ^+^)
AgNPs-3MPS + Co^2+^ (1.0 ppm)	Ag3d_5/2_	368.2	1.3	80%	Ag(0)
	Ag3d_5/2_	369.3	“	20%	Ag(δ^+^)

* I ratios = I_peak_/I_tot_ signal for a selected element.

**Table 3:** HR-XPS S2p core levels data collected on silver nanoparticles (NPs) stabilized by 3MPS (BE, FWHM, atomic ratio, and assignments) and AgNPs-3MPS in presence of nickel and cobalt ions (1.0 ppm).

Sample	Signal	BE (eV)	FWHM (eV)	* I Ratio	** Assignments
AgNPs-3MPS	S2p3/2	161.2	1.5	54%	RS-Ag
	S2p3/2	168.0	“	46%	SO_3_^−^Na^+^
AgNPs-3MPS + Ni^2+^ (1.0 ppm)	S2p3/2	162.2	1.5	30%	RS-Ag
	S2p3/2	163.2	“	26%	RS-H
	S2p3/2	168.2	“	16%	SO_3_^−^Na^+^
	S2p3/2	168.8	“	28%	SO_3_^−^-M
AgNPs-3MPS + Co^2+^ (1.0 ppm)	S2p3/2	162.3	1.7	22%	RS-Ag
	S2p3/2	163.0	“	24%	RS-H
	S2p3/2	168.3	“	32%	SO_3_^−^Na^+^
	S2p3/2	168.8	“	22%	SO_3_^−^-M

* I ratios = I_peak_/I_tot_ signal for a selected element. ** M = Ni^2+^; Co^2+^.

**Table 4:** HR-XPS Ni2p_3/2_ and Co2p_3/2_ metal core levels data collected on silver NPs stabilized by 3MPS in presence of nickel and cobalt ions (BE, FWHM, and assignments).

Sample	BE (eV)	FWHM (eV)	Assignments
AgNPs-3MPS + Ni^2+^ (1.0 ppm)	856.2	4.3	Ni^2+^ Coordination Compounds
	861.9	6.3	Satellite Structure
AgNPs-3MPS + Co^2+^ (1.0 ppm)	782.3	4.6	Co^2+^ Coordination Compounds
	787.2	8.3	Satellite Structure

## Supplementary Materials

The following are available online at http://www.mdpi.com/2079-4991/8/7/488/s1, Figure S1: Fitting curves with a sigmoidal Richards for Ni^2+^ (A) and Co^2+^ (B); Figure S2: DLS measurements of AgNPs-3MPS: (a) <2R_H_> = 8.5 ± 2.6 nm; (b) ζ potential = −42 ± 5 (mV); Figure S3: DLS measurements of AgNPs-3MPS in presence of 1 ppm of Ni^2+^: (a) <2R_H_> = 43 ± 4 nm; (b) ζ potential = −27 ± 10 (mV); Figure S4: DLS measurements of AgNPs-3MPS in presence of 1 ppm of Co^2+^: (a) <2R_H_> = 76 ± 9 nm; (b) ζ potential = −22 ± 15 (mV); Figure S5: FTIR spectra in the 4000–600 cm^−1^ range (with a 2500–2000 cm^−1^ break) of pristine 3MPS (black line) and AgNP-3MPS after interaction with Ni^2+^ ions (red line); Figure S6: TEM image of AgNPs-3MPS showing the good uniformity and shape of the nanoparticles; Figure S7: High resolution TEM image of one nanoparticle showing its crystallinity. The lattice parameter estimated by the image is 0.24 nm as shown in the inset; Figure S8: Ag3d XPS spectra.
