# Supplementary material for: Plasmonic Sensor Based on Interaction between Silver Nanoparticles and Ni2+ or Co2+ in Water
Source: Nanomaterials (Basel). 2018 Jul 2;8(7):488. doi: 10.3390/nano8070488 (PMC6070780; doi:10.3390/nano8070488)

Supporting Information

Plasmonic sensor based on interaction between silver nanoparticles and Ni^{2+} or Co^{2+} in water

Federico Mochi [1,2], Luca Burratti [1], Ilaria Fratoddi [3], Iole Venditti [4,*], Chiara Battocchio [4], Laura Carlini [4], Giovanna Iucci [4], Mauro Casalboni [1,2], Fabio De Matteis [1,2], Stefano Casciardi [5], Silvia Nappini [6], Igor Pis [7] and Paolo Prosposito [1,2,*]

[1] Department of Industrial Engineering and INSTM, University of Rome, Tor Vergata, via del Politecnico 1, 00133, Rome, Italy; paolo.prosposito@uniroma2.it, federico.mochi@uniroma2.it, luca.burratti@uniroma2.it

[2] Center for Regenerative Medicine, University of Rome Tor Vergata, Via Montpellier 1, 00133, Rome, Italy; casalboni@uniroma2.it, fabio.dematteis@uniroma2.it

[3] Department of Chemistry, University of Rome Sapienza, Rome, P.le A. Moro 5, 00187, Italy; ilaria.fratoddi@uniroma1.it

[4] Department of Sciences, Roma Tre University of Rome Via della Vasca Navale 79, 00146 , Italy; iole.venditti@uniroma3.it; chiara.battocchio@uniroma3.it ; laura.carlini@uniroma3.it ; giovanna.iucci@uniroma3.it

[5] National Institute for Insurance against Accidents at Work (INAIL), Department of Occupational and Environmental Medicine, Epidemiology and Hygiene, 00078 Monte Porzio Catone, Italy; s.casciardi@inail.it

[6] IOM-CNR Laboratorio TASC, SS 14, km 163,5 Basovizza, I-34149 Trieste, Italy; nappini@iom.cnr.it

[7] Elettra-Sincrotrone Trieste S.C.p.A., SS 14, km 163,5 Basovizza, I-34149 Trieste, Italy; igor.pis@elettra.eu

* Correspondence: paolo.prosposito@uniroma2.it Tel.: +39 0672594115; iole.venditti@uniroma3.it Tel.: +390657333388

SI-Figure 1. Fitting curves with a sigmoidal Richards for Ni^{2+} (A) and Co^{2+} (B)

Equation: Sigmoidal Richards

y = a*(1 + (d-1)*exp(-k*(x-xc)))^(1/(1-d))

(A)

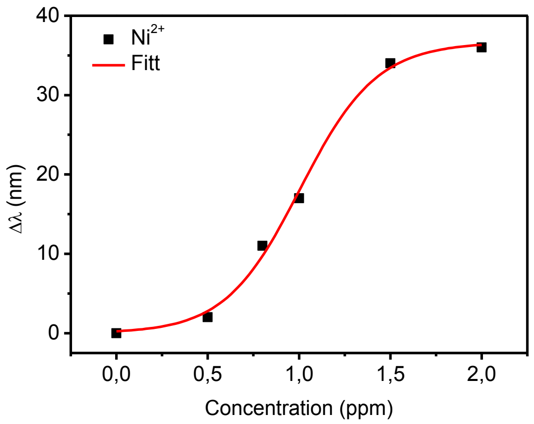

Reduced Chi-Sqr	1,7626		
Adj. R-Square	0,99265		
		Value	Standard Error
DL	a	36,66619	1,69105
DL	xc	1,00389	0,07418
DL	d	1,93313	0,84486
DL	k	4,71923	1,83344

(B)

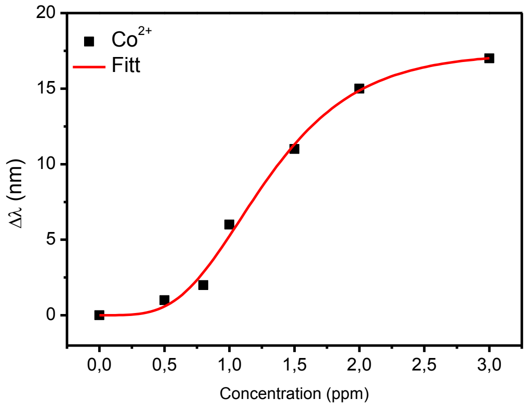

Reduced Chi-Sqr	0,51917		
Adj. R-Square	0,98925		
		Value	Standard Error
DL	a	17,3625	0,98184
DL	xc	1,08073	0,1617
DL	d	0,97485	0,57236
DL	k	2,03115	0,69884

Si- Figure 2. DLS measurements of AgNPs-3MPS : a) <2R_H> = 8.5 ± 2.6 nm; b) ζ potential = - 42 ± 5 (mV)

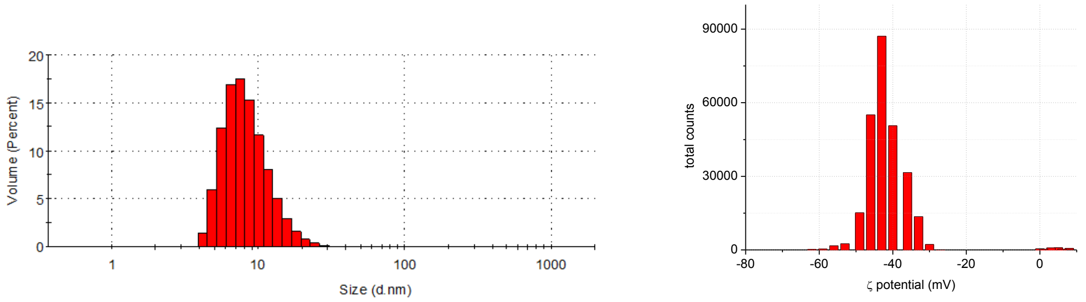

SI-Figure 3. DLS measurements of AgNPs-3MPS in presence of 1 ppm of Ni^{2+}: a) <2R_H> = 43 ± 4 nm; b) ζ potential = -27 ± 10 (mV)

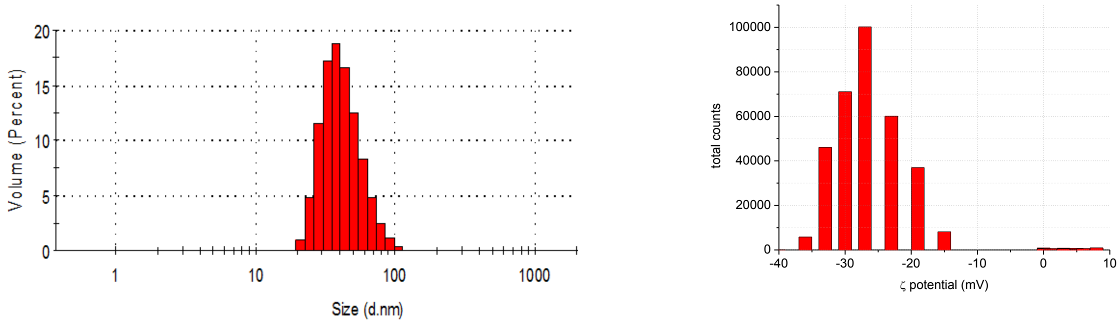

SI-Figure 4. DLS measurements of AgNPs-3MPS in presence of 1 ppm of Co^{2+}: a) <2R_H> = 76 ± 9 nm; b) ζ potential = -22 ± 15 (mV)

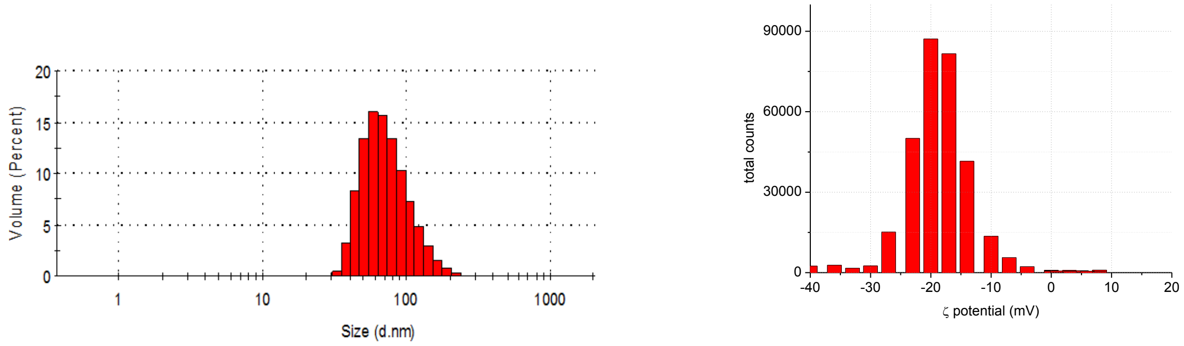

SI-Figure 5. FTIR spectra in the 4000-600 cm^{-1} range (with a 2500-2000 cm^{-1} break) of pristine 3MPS (black line) and AgNP-3MPS after interaction with Ni^{2+} ions (red line).

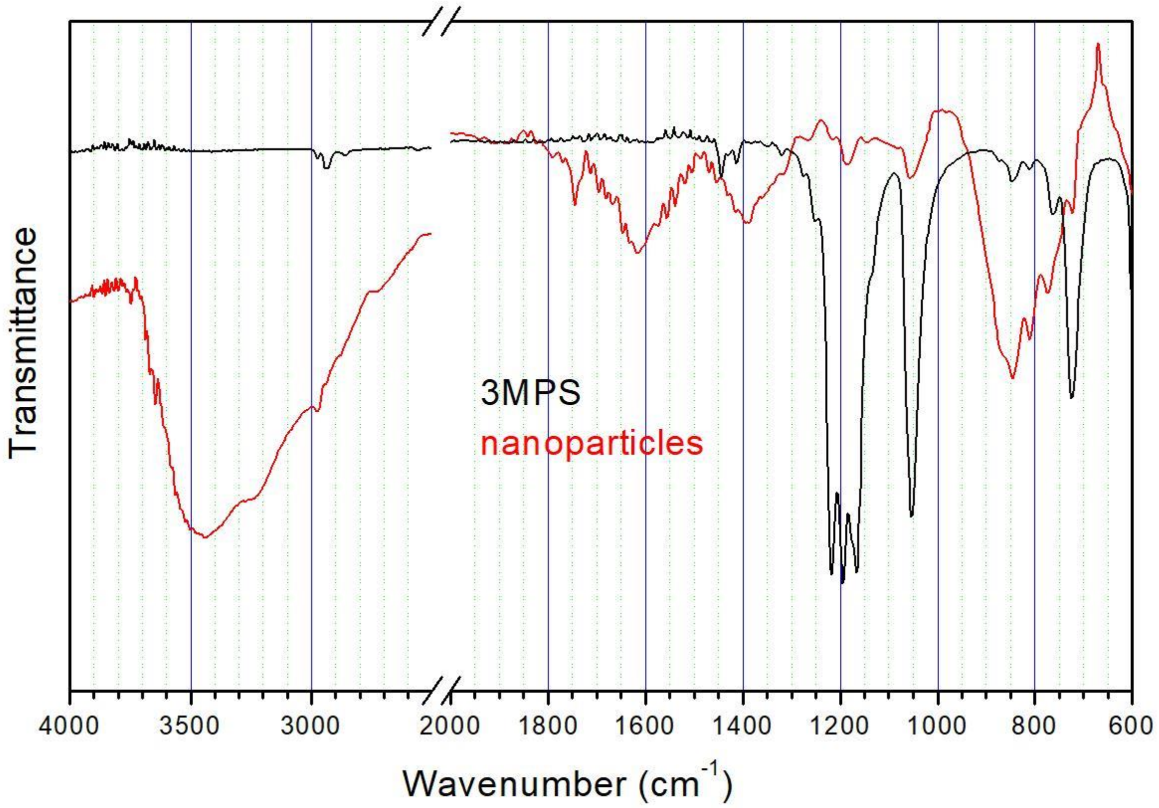

SI-Figure 6. TEM image of AgNPs-3MPS showing the good uniformity and shape of the nanoparticles.

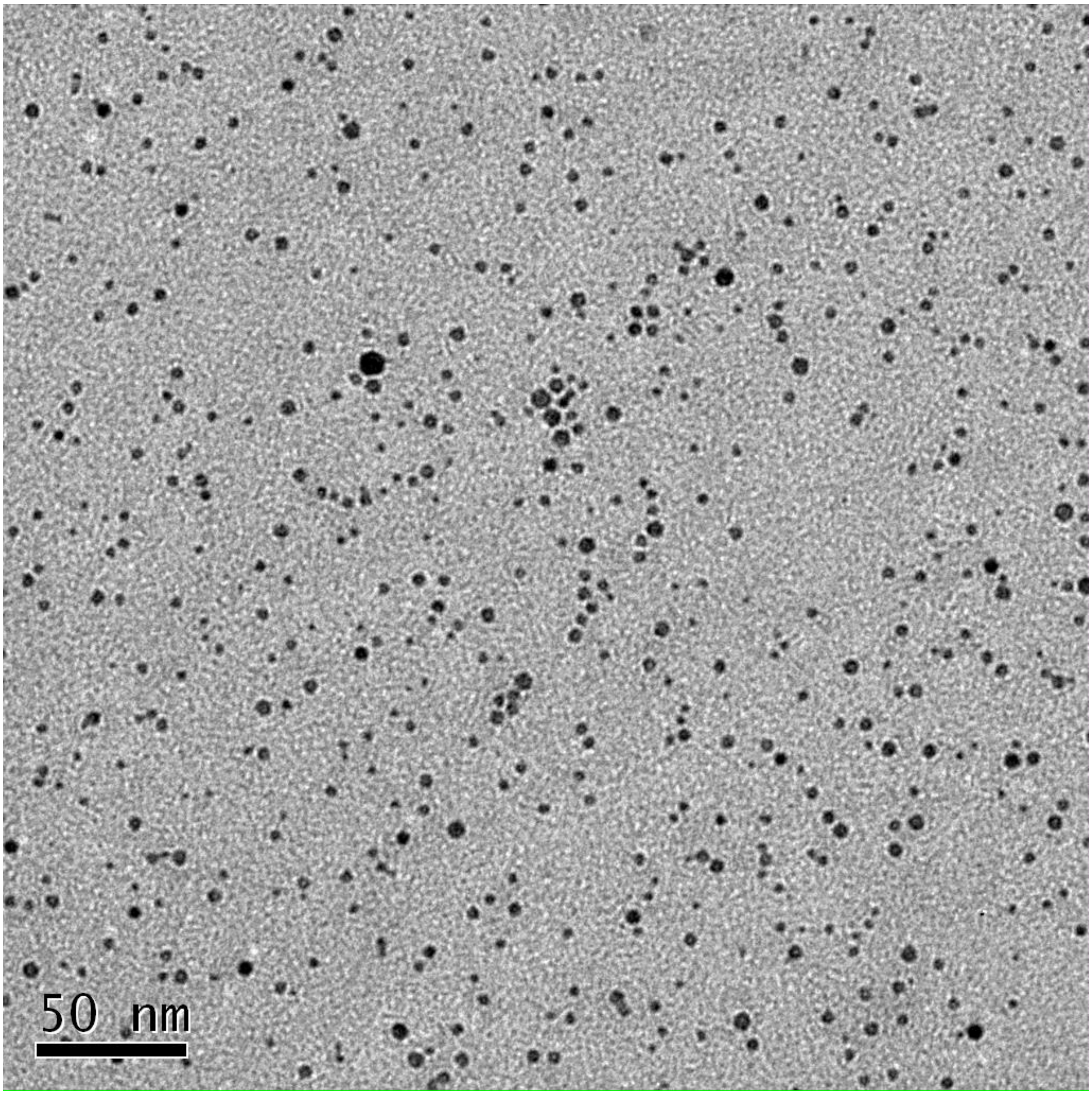

SI-Figure 7. High resolution TEM image of one nanoparticle showing its crystallinity. The lattice parameter estimated by the image is 0.24 nm as shown in the inset.

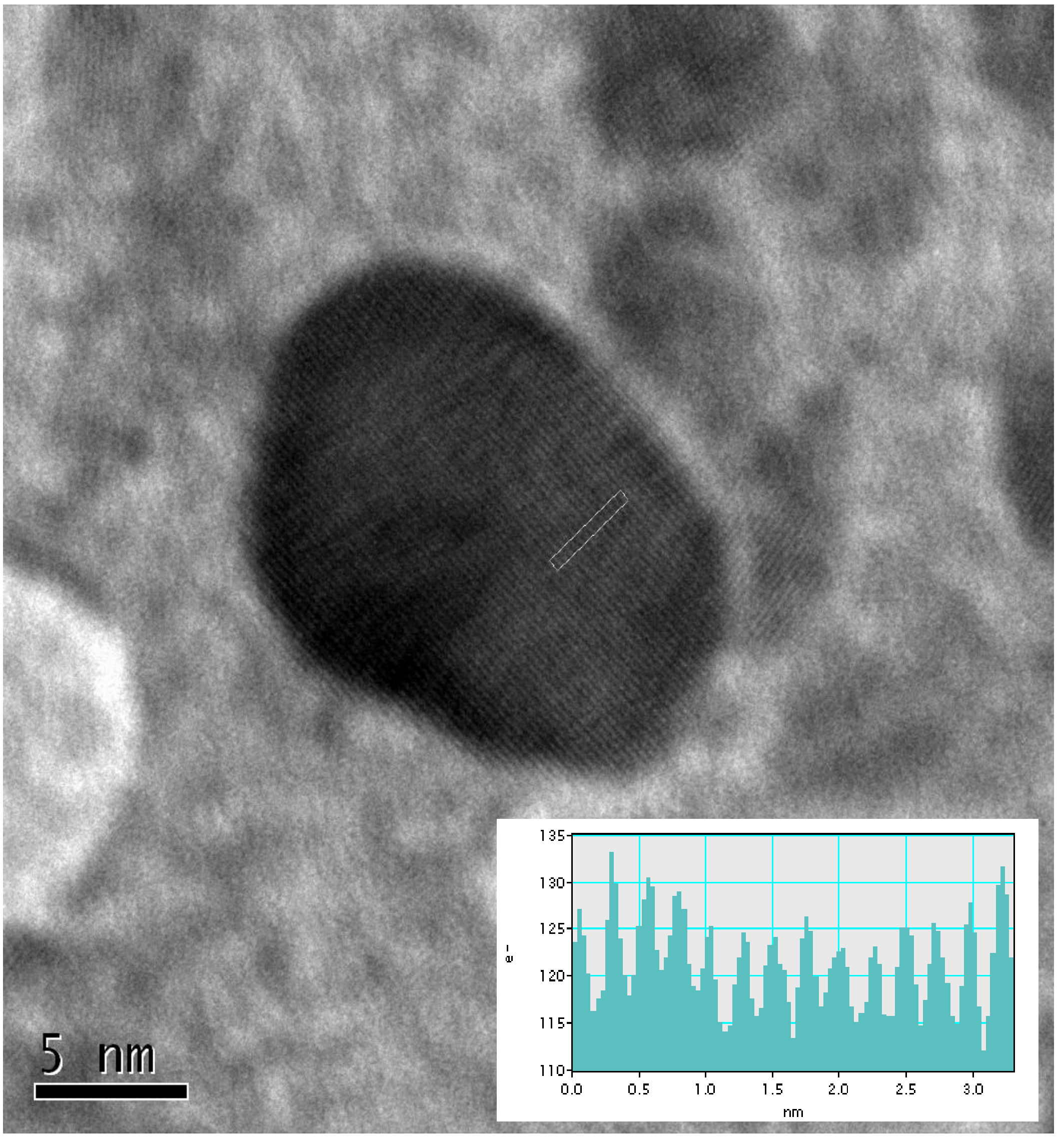

SI-Figure 8. Ag3d XPS spectra

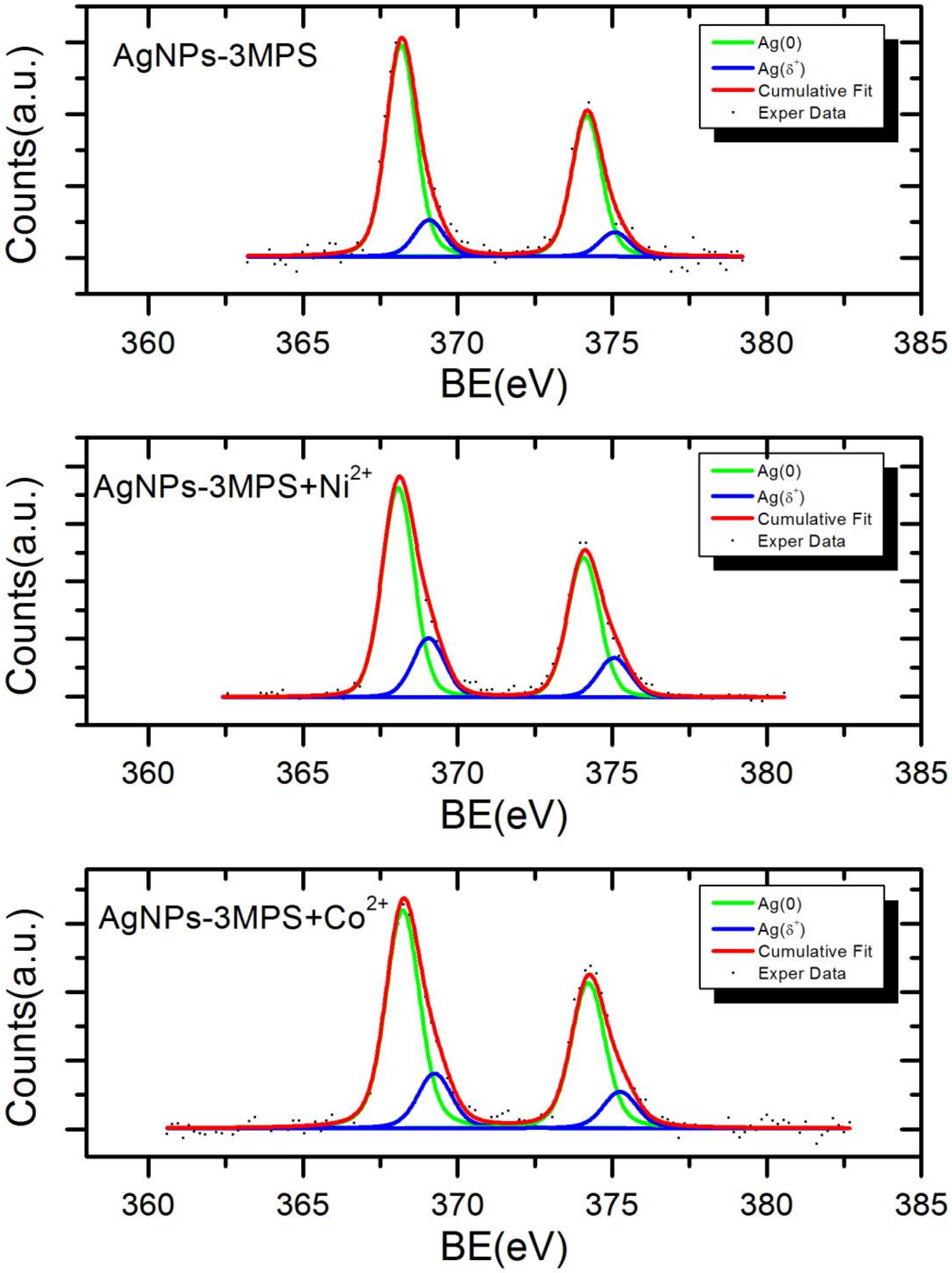

Supplement: Supplementary file 1 [file nanomaterials-08-00488-s001.pdf]
